# Comprehensive Analysis of Inflammatory Immune Mediators in Vitreoretinal Diseases

**DOI:** 10.1371/journal.pone.0008158

**Published:** 2009-12-04

**Authors:** Takeru Yoshimura, Koh-Hei Sonoda, Mika Sugahara, Yasutaka Mochizuki, Hiroshi Enaida, Yuji Oshima, Akifumi Ueno, Yasuaki Hata, Hiroki Yoshida, Tatsuro Ishibashi

**Affiliations:** 1 Department of Ophthalmology, Graduate School of Medical Sciences, Kyushu University, Fukuoka, Japan; 2 Department of Biomolecular Sciences, Faculty of Medicine, Saga University, Nabeshima, Saga, Japan; Universidade Federal do Rio de Janeiro (UFRJ), Brazil

## Abstract

Inflammation affects the formation and the progression of various vitreoretinal diseases. We performed a comprehensive analysis of inflammatory immune mediators in the vitreous fluids from total of 345 patients with diabetic macular edema (DME, n = 92), proliferative diabetic retinopathy (PDR, n = 147), branch retinal vein occlusion (BRVO, n = 30), central retinal vein occlusion (CRVO, n = 13) and rhegmatogenous retinal detachment (RRD, n = 63). As a control, we selected a total of 83 patients with either idiopathic macular hole (MH) or idiopathic epiretinal membrane (ERM) that were free of major pathogenic intraocular changes, such as ischemic retina and proliferative membranes. The concentrations of 20 soluble factors (nine cytokines, six chemokines, and five growth factors) were measured simultaneously by multiplex bead analysis system. Out of 20 soluble factors, three factors: interleukin-6 (IL-6), interleukin-8 (IL-8), and monocyte chemoattractant protein-1 (MCP-1) were significantly elevated in all groups of vitreoretinal diseases (DME, PDR, BRVO, CRVO, and RRD) compared with control group. According to the correlation analysis in the individual patient's level, these three factors that were simultaneously increased, did not show any independent upregulation in all the examined diseases. Vascular endothelial growth factor (VEGF) was significantly elevated in patients with PDR and CRVO. In PDR patients, the elevation of VEGF was significantly correlated with the three factors: IL-6, IL-8, and MCP-1, while no significant correlation was observed in CRVO patients. In conclusion, multiplex bead system enabled a comprehensive soluble factor analysis in vitreous fluid derived from variety of patients. Major three factors: IL-6, IL-8, and MCP-1 were strongly correlated with each other indicating a common pathway involved in inflammation process in vitreoretinal diseases.

## Introduction

Vitreoretinal diseases, such as diabetic retinopathy (DR), retinal vein occlusion (RVO), and retinal detachment (RD) have a poor visual prognosis. Although these diseases have variety of etiology, their pathogenic retinal changes (including angiogenesis and fibrosis) cause local inflammation. In fact, infiltration of leukocytes into the choroid, retina, and vitreous is observed in various vitreoretinal disorders, including PDR [1], proliferative vitreoretinopathy (PVR) [2], as well as in obvious inflammatory ocular diseases such as endophthalmitis and uveitis. Even though such leukocyte infiltration may be only a secondary event, it damages retinal tissues.

During inflammation a variety of soluble factors are secreted into the vitreous cavity (posterior chamber of the eye) and their concentrations may reflect visual prognosis. Cytokines, which usually serve as signals between neighboring cells, are involved in essentially every important biological process, including cell proliferation, inflammation, immunity, migration, fibrosis, tissue repair, and angiogenesis [3], [4]. Chemokines are multifunctional mediators that can direct the recruitment of leukocytes to sites of inflammation, promote the process, enhance immune responses, and promote stem cell survival, development, and homeostasis [5]. Recently, it has been demonstrated that chemokines play a pivotal role in mediating angiogenesis and fibrosis as well [6], [7].

DR is one of the most severe complication of diabetes mellitus and a leading cause of blindness. DR can further be divided into non-proliferative diabetic retinopathy (NPDR) and PDR. NPDR causes central vision loss when it induces DME. PDR is the most advanced stage of DR, which is characterized by retinal neovasculization. The pathology of PDR includes vitreous hemorrhage, formation of fibrous peri-retinal tissue comprising neovascular blood vessels, tractional retinal detachment and total vision loss as the final stage. Various mechanisms play a role in the pathogenesis of DR, including the disruption of blood-retinal barrier, alterations to capillary vessel walls, synthesis of growth factors and nitric oxide, disruption of connective tissue by matrix metalloproteinases, and activation of various immune mechanisms.

RVO is another common retinal vascular disorder and a common cause of visual impairment. Between the two types of RVO, BRVO is more common than CRVO. The pathogenesis of CRVO is multifactorial while BRVO is believed to be driven by a combination of three primary mechanisms: compression of the vein at the arteriovenous crossing, degenerative changes of the vessel wall, and abnormal hematological factors [8].

RRD is defined as the separation of the neurosensory retina from subjacent retinal pigment epithelium (RPE) caused by penetration of fluids into the subretinal space via one or more full-thickness retinal breaks. Initial detachment may be local, but without rapid treatment the entire retina may detach, which may lead to vision loss and blindness. Lewandowska-Furmanik et al. suggested the involvement of the immune system in pathogenesis of RRD following the detection of some cytokine concentrations in subretinal fluid of 36 RRD patients [9].

Aiello et al. reported VEGF was detected from ocular fluid with diabetic retinopathy and other retinal disorders [10]: there are other reports measuring multiple soluble factors in samples from different vitreoretinal disorders using conventional enzyme-linked immunosorbent assay (ELISA) [11]-[16]. However, the examination of complex patterns of these factors in human vitreoretinal diseases has been limited by the small number of vitreous samples available from each patient. Recently, a particle-based flow cytometric analysis method has been established to improve the conventional method and overcome many of these limitations [17]. Two recent studies demonstrated the analysis of vitreous inflammatory mediators by multiplex bead analysis in 58 patients with several vitreoretinal disorders and 32 patients with diabetic patients [18], [19]. Djoba Siawaya and colleagues [20] compared between the multiplex assays based on Luminex technology and established ELISA technique. Their conclusion was that currently the most appropriate use for the Luminex technology is as a screening tool.

In this study we report the use of Luminex technique for analysis of complex network of immune mediators in vitreous humor and the relations between them; we examined a profile of immune mediators in 345 eyes from patients undergoing vitrectomy. Moreover, the correlations between several immune mediators related to different vitreoretinal diseases were determined. Finding patterns in expression of inflammatory cytokines specific to a particular disease can substantially contribute to the understanding of the basic mechanism of this disease and consequently to the development of a targeted therapy.

## Results

### Detection of Soluble Factors in Vitreous Fluids from Patients

The bibliography of enrolled patients was shown in Table 1. The investigated inflammatory mediators were categorized into three groups: (1) nine cytokines: IL-1β, IL-2, IL-4, IL-5, IL-6, IL-10, IL-17, interferon-γ (IFN-γ), tumor necrosis factor-α (TNF-α) (Table 2); (2) six chemokines: IL-8, eotaxin, MCP-1/CCL2, macrophage inflammatory protein-1α (MIP-1α), MIP-1β, and regulated on activation, normal T cell expressed and secreted (RANTES)/CCL5 (Table 3); and (3) five growth factors: epidermal growth factor (EGF), VEGF, basic FGF, granulocyte colony-stimulating factor (G-CSF) and granulocyte-macrophage colony-stimulating factor (GM-CSF) (Table 4). We found that four out of twenty soluble factors: IL-6, IL-8, MCP-1, and VEGF were predominantly detected in vitreous fluids from patients (Table 2-4). In addition, MIP-1β, G-CSF, and GM-CSF were detected in limited number of patients. For instance, we found one CRVO patient with extremely high concentration of these four cytokines (IL-6: 11103 pg/ml, IL-8: 6821 pg/ml, MCP-1: 15403 pg/ml, VEGF: 11737 pg/ml) that showed extra high concentrations of G-CSF (467.6 pg/ml) and GM-CSF (1036 pg/ml).

**Table 1:** The bibliography of enrolled patients (age: mean±SD).

	female	male	total	
**control**	n = 57 (65.7±8.2)	n = 26 (67.8±6.8)	n = 83 (66.4±7.8)	
**DME**	n = 46 (63.9±7.4)	n = 46 (62.4±9.7)	n = 92 (63.0±8.6)	DME/PDR: p<0.0001§
**PDR**	n = 43 (56.3±13.6)	n = 104 (55.6±12.1)	n = 147 (55.8±12.5)	
**BRVO**	n = 17 (70.0±10.6)	n = 13 (69.2±11.2)	n = 30 (69.7±10.7)	BRVO/CRVO: p = 0.5969§
**CRVO**	n = 7 (69.6±11.0)	n = 6 (74.0±11.1)	n = 13 (71.6±10.8)	
**RRD**	n = 28 (62.3±12.0)	n = 35 (60.6±9.3)	n = 63 (61.4±10.5)	
P value	p = 0.0003*	p<0.0001*	p<0.0001*	

* Kruskal-Wallis test,

§ Mann-Whitney U test.

**Table 2:** Concentrations of cytokines in the vitreous cavity.

	IL-1β	IL-2	IL-4	IL-5	IL-6	IL-10	IL-17	IFN-γ	TNF-α
**Control**	<150	<60	<50	<30	12.1(<30–206.8)	<50	<100	<50	<100
**DME**	<150	<60	<50	<30	174(<30–1152)	<50	<100	<50	<100
**PDR**	<150	<60	<50	<30	330.1(<30–8630)	<50	<100	<50	<100
**BRVO**	<150	<60	<50	<30	65.6(<30–326)	<50	<100	<50	<100
**CRVO**	<150	<60	<50	<30	985.4(<30–11103)	<50	<100	<50	<100
**RRD**	<150	<60	<50	<30	701.6(<30–15381)	<50	<100	<50	<100

Values are given as the mean (range) in pg/ml, with the detection limit for each mediator.

**Table 3 pone-0008158-t003:** Concentrations of chemokines in the vitreous cavity.

	IL-8	eotaxin	MCP-1	MIP-1α	MIP-1β	RANTES
**control**	28.9(<30–1538)	<50	100.8(<100–2146.9)	<100	<100	<150
**DME**	258(<30–5522)	<50	1590.1(<100–38394)	<100	<100	<150
**PDR**	394.2(<30–11700)	<50	1665(<100–31099.5)	<100	<100	<150
**BRVO**	473.2(<30–7728)	<50	439.1(<100–2895)	<100	<100	<150
**CRVO**	1027(<30–6822)	<50	2906(195–15045)	<100	<100	<150
**RRD**	285.9(<30–4757)	<50	3944(<100–35811)	<100	<100	<150

Values are given as the mean (range) in pg/ml, with the detection limit for each mediator.

**Table 4:** Concentrations of growth factors in the vitreous cavity.

	EGF	VEGF	bFGF	G-CSF	GM-CSF
**control**	<150	111.0(<150–642.4)	<150	<150	<150
**DME**	<150	175.2(<150–1500.8)	<150	<150	<150
**PDR**	<150	545.7(<150–7900.0)	<150	<150	<150
**BRVO**	<150	167.4(<150–1977.0)	<150	<150	<150
**CRVO**	<150	1635.0(<150–11737)	<150	<150	<150
**RRD**	<150	77.4(<150–712)	<150	<150	<150

Values are given as the mean (range) in pg/ml, with the detection limit for each mediator.

The concentrations of IL-1β, IL-2, IL-4, IL-5, IL-10, IL-17, IFN-γ, TNF-α, eotaxin, MIP-1α, RANTES, EGF and bFGF were under the detection level in all the examined samples (the minimal detectable concentration is listed in Table 2–4). Technical issues were excluded as control recombinant proteins were detected (data not shown). Moreover, concentrations of soluble factors (IFN-γ, TNF-α, and IL-2) in aqueous humor from acute uveitis patients (some samples were actually measured on the same plate in this study series) were detected [21], in accordance with previous findings [22]. In addition extremely high concentrations of IL-10 were detected in vitreous fluids from intraocular malignant lymphoma patients in this system (data not shown), in support with previous findings [23].

### IL-6, IL-8 and MCP-1 Were Increased in All Examined Diseases, but VEGF Was Increased in PDR and CRVO Patients Only

**Figure 1:**
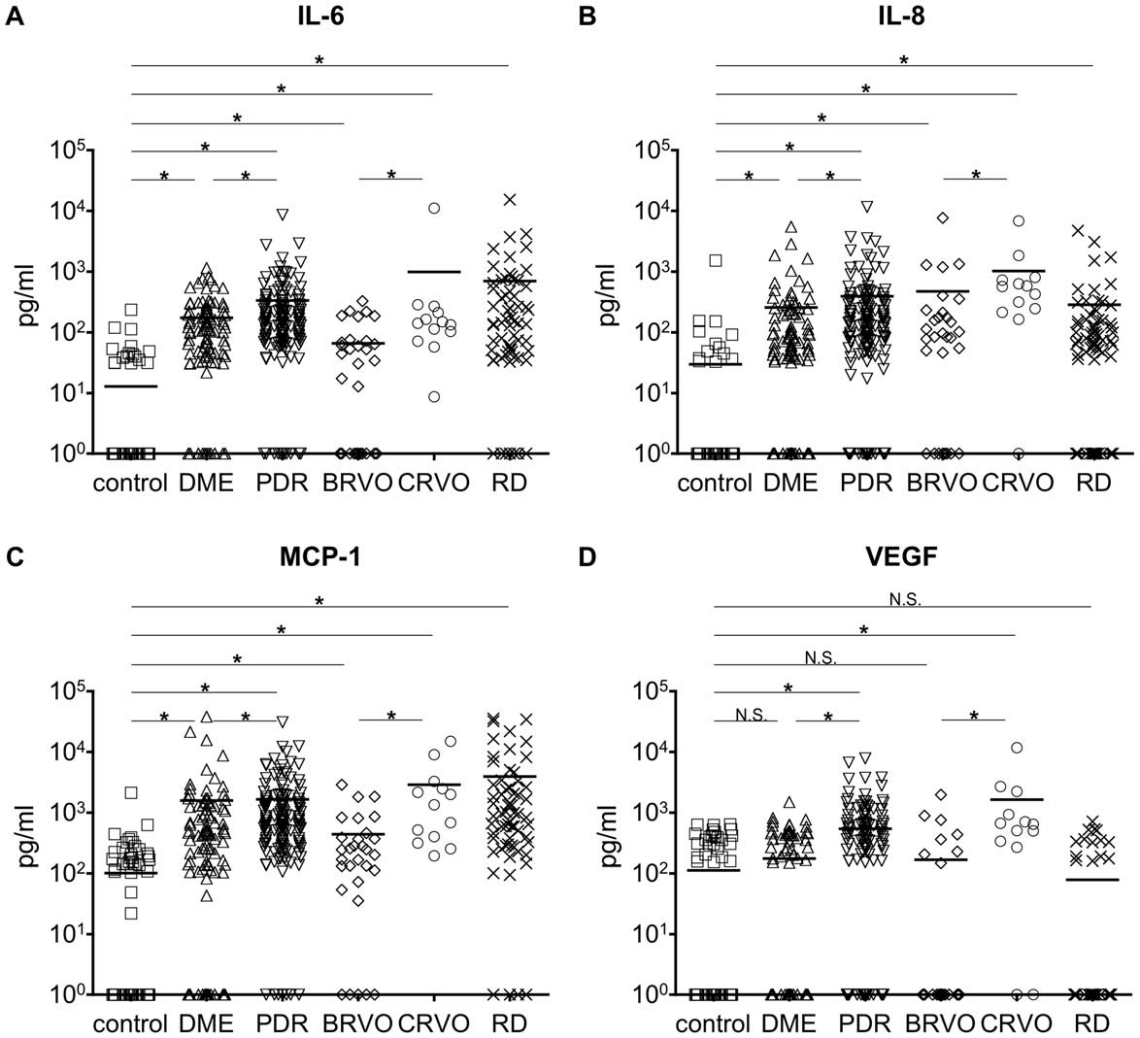
Detection of soluble factors in vitreous fluids from patients. (A) IL-6, (B) IL-8, (C) MCP-1, and (D) VEGF levels in vitreous fluid of control patients and patients with DME, PDR, BRVO, CRVO, and RRD. The ordinate showed the concentrations of soluble factors in the log scale, bars represent the mean value of each group. * *P*<0.05, N.S.: not significant.

Since the four factors (IL-6, IL-8, MCP-1 and VEGF) could be detected in the majority of the patients, we decided to further study these factors. In Figure 1, every individual dot represents a measured concentration which is plotted in log scale (y axis) and bars represent the mean value of each group. Compared with control subjects (either ERM or MH), the concentrations of IL-6 (Figure 1A), IL-8 (Figure 1B), and MCP-1 (Figure 1C) were significantly higher in patients with DME, PDR, BRVO, CRVO, and RRD. VEGF levels were significantly higher in samples from patients with either PDR or CRVO than in control(Figure 1D), but not in samples from DME nor BRVO despite the same category of disease used (DR and RVO, respectively). RD patients did not show any elevated levels of VEGF.

The concentrations of all four factors, IL-6, IL-8, MCP-1 and VEGF, were significantly high within the same disease category, where in PDR the level was higher than DME, and in CRVO these factors level were higher than in BRVO (Figure 1A–D).

### IL-6, IL-8 and MCP-1 Are Mutually Increased in All Analyzed Vitreoretinal Diseases

**Figure 2:**
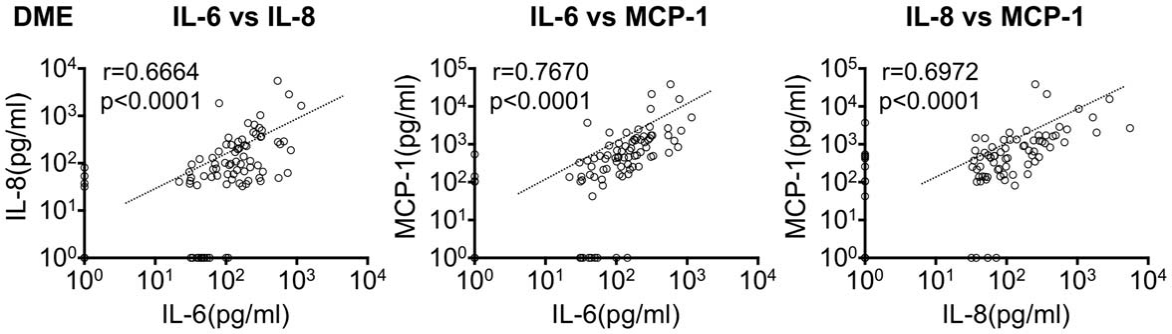
IL-6, IL-8 and MCP-1 were mutually increased in all examined vitreoretinal diseases. As an example, Spearman's correlation analysis of DME are shown (n = 92). In each disease, total of three combinations (IL-6/IL-8, IL-6/MCP-1, and IL-8/MCP-1) were created, and calculated p- and r-values which indicating the accuracy of correlation.

**Figure 3:**
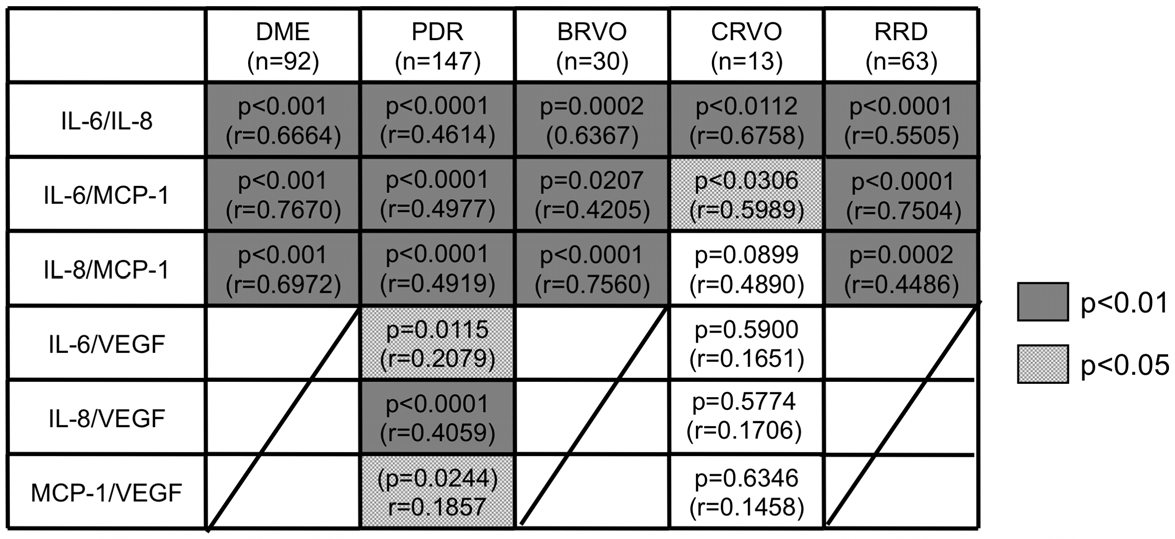
Summary of correlation analysis. The combination of two factors was calculated for each individual disease and listed in the ordinate.

Since the three factors: IL-6, IL-8 and MCP-1 were commonly upregulated in all the examined five diseases (DME, PDR, BRVO, CRVO, and RRD), a Spearman's correlation analysis was performed between the three factors. In each disease, we analyzed total of three combinations (two factors out of three), represented in dots, and calculated p- and r-values which indicate the accuracy of the correlation. An example is the correlation shown in DME data where the dots are placed along with the median line, p-values are less than 0.05, and r-values are high in all three combinations (IL-6/IL-8, IL-6/MCP-1 and IL-8/MCP-1) (Figure 2). This setting means that the three factors are correlated with each other in DME patients. The results of p-values and r-values are shown in Figure 3 (upper three lines). All three combinations of IL-6/IL-8, IL-6/MCP-1 and IL-8/MCP-1 in the studied groups showed significant correlations (p<0.05) except from IL-8/MCP-1 in CRVO (Figure 3, upper three lines).

### VEGF Independently Contribute to the Pathogenic Process of PDR and CRVO

**Figure 4:**
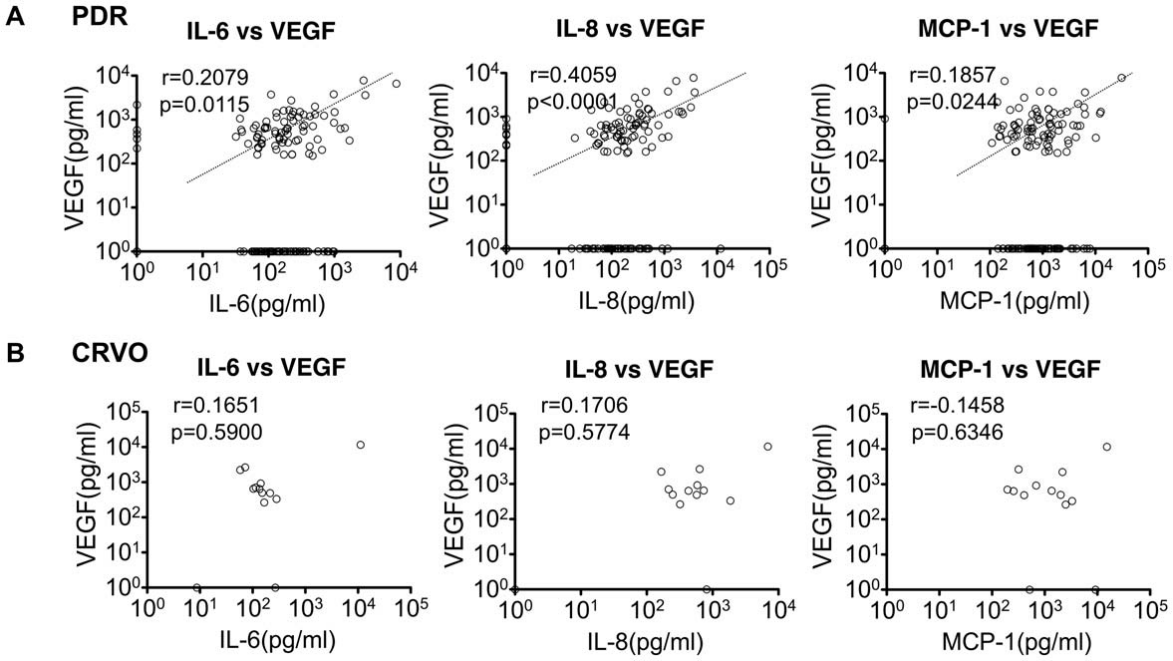
VEGF independently contribute to the pathogenic process of PDR and CRVO. Spearman's correlation analysis between VEGF and IL-6/IL-8/MCP-1 in patients with PDR (n = 147) or CRVO (n = 13) are shown. In each disease, total of three combinations (VEGF/IL-6, VEGF/IL-8, and VEGF/MCP-1) were analyzed, and calculated p- and r-values which indicating the accuracy of correlation.

Since VEGF was significantly increased in PDR and CRVO patients (Figure 1), the correlation between VEGF and the three factors: IL-6, IL-8, and MCP-1 in PDR and CRVO were investigated. Figure 3 shows the result of six combinations including VEGF in PDR (Figure 4A) and CRVO (Figure 4B). The p-values and r-values are summarized in Figure 3 (lower three lines). In PDR patients, the elevation of VEGF was significantly correlated with the other three factors, while no significant correlation was observed in CRVO patients (Figure 3, lower three lines). Unlike the three factors: IL-6, IL-8 and MCP-1, VEGF was not a common factor, but may independently play a role in the pathologic process in PDR and CRVO.

### Serum IL-6 and VEGF Concentrations Are Not Significantly Related with Vitreous Concentrations

**Figure 5:**
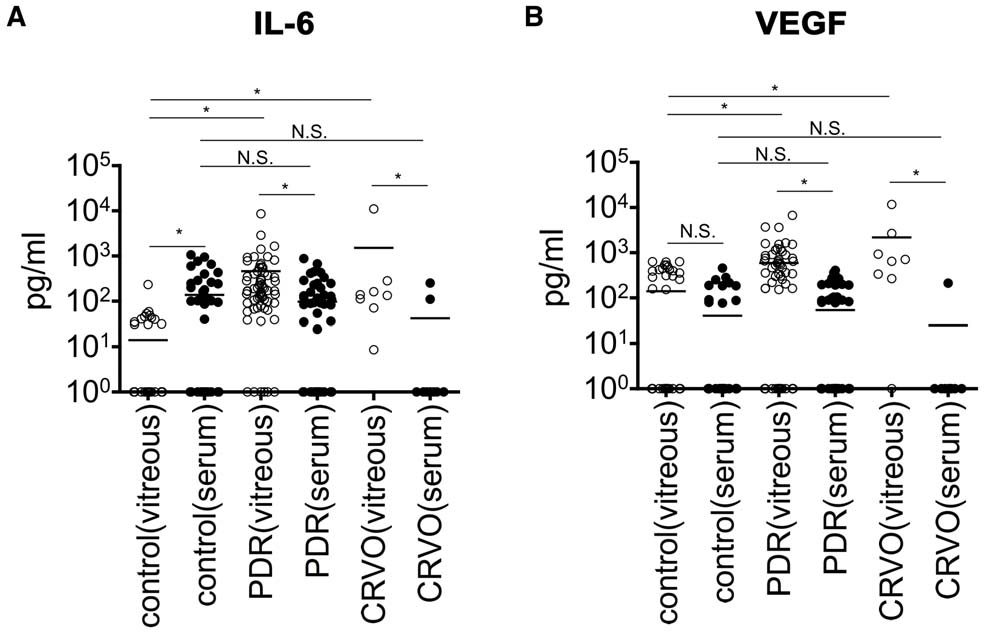
Serum IL-6 and VEGF concentrations are not significantly related with vitreous concentrations. Shown are comparison of (A) IL-6, and (B) VEGF levels in vitreous and in serum of patients with control (n = 53), PDR (n = 66) and CRVO (n = 8). The ordinate showed the concentrations of soluble factors in the log scale, bars represent the mean value of each group. Bars represent the mean value of each group. * *P*<0.01, N.S.: not significant.

Because vitreoretinal diseases examined in this study develop neovasculization in the eye, it is likely to expect that mediators in the circulation are easily and passively enter the vitreous. Therefore, we decided to measure the proteins in serum in PDR and CRVO groups as neovascularized eye diseases. Based on our findings, we measured IL-6, as a representative inflammatory marker, and VEGF concentrations in serum from control patients and patients with highly angiogenic diseases using standard ELISA technique. All serum samples we could have and vitreous samples which correspond to each patient were statistically analyzed (control: n = 53, PDR: n = 66, CRVO: n = 8; Table 5 and Figure 5). Even in the reduced numbers, we confirmed vitreous IL-6/VEGF concentrations were significantly increased in PDR/CRVO patients than control. However, serum IL-6/VEGF concentrations were not increased in PDR/CRVO patients (Figure 5). The data clearly indicate that vitreous soluble factors are mainly from ocular tissues.

**Table 5:** Concentrations of IL-6 and VEGF in the vitreous and serum.

	vitreous IL-6	serum IL-6	vitreous VEGF	serum VEGF
**control**	13.2(<30–237.1)	139.4(<47.0–1070)	139.6(<150–642.4)	47.8(<156.5–459.4)
**PDR**	463.3(<30–8630)	97.0(<47.0–875.3)	584(<150–6700)	53.1(<156.5–407.6)
**CRVO**	1502(<30–11104)	40.6(<47.0–254.0)	2168(<150–11737)	23.8(<156.5–214.4)

Values are given as the mean (range) in pg/ml, with the detection limit for each mediator.

## Discussion

Inflammatory processes have been considered to be critical in vitreoretinal diseases [24]-[26]. The concentrations of inflammatory soluble factors might not necessarily reflect a pathogenic process, especially the microenvironments inside the retina. However, secreted factors in the vitreous cavity appear to be associated with pathological processes. Analyzing these factors can provide new insights relating to the biological mechanism of the disease and to the design of a therapeutic strategy: in fact, there are some reports suggesting intravitreal triamcinolone acetonide as a drug with an anti-inflammatory property [27]–[29]. Many studies describing the analysis of soluble factor profiles in vitreous fluids in a particular disease have been performed on limited numbers of patients [10]–[16]. We herein collected 345 samples from five different diseases and performed comprehensive analysis of 20 factors for the first time.

**Figure 6:**
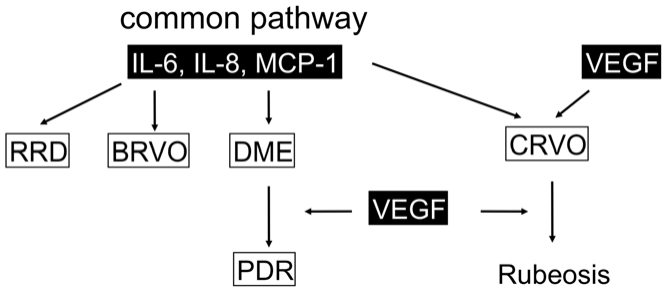
Possible contribution of major four factors: IL-6, IL-8, MCP-1 and VEGF in vitreoretinal diseases. A scheme illustrates the relations between the different mediators in samples from the different vitreoretinal diseases: IL-6, IL-8, and MCP-1 in the vitreous cavity can increase vascular permeability that causes DME, but an additional retinal ischemia led to increased VEGF production that play an important role in the progression of DME to PDR. At the same time, high VEGF levels may be initially produced by the sudden profound retinal injury, and then induced major three factors, frequently resulted in rubeosis iriditis.

One of our most important finding is that the major three factors: IL-6, IL-8, and MCP-1 were commonly upregulated in all the examined diseases (Figure 1) and were correlated with each other without any independent change (Figure 3). The p-value of IL-8/MCP-1 in CRVO which was “not significant”, (P = 0.0899), but this may be due to the small number of samples (n = 13) used. In general, the three factors (IL-6, IL-8, and MCP-1) were correlated with each other and increased synchronizing. This high correlation between the three factors indicates a common pathway is involved in the formation various vitreoretinal disorders (Figure 3, 6); IL-6 is a multifunctional cytokine that may indirectly cause an increase of vascular permeability by inducing the expression of VEGF [30] or alternatively may directly increase endothelial cell permeability [31]. IL-8 is produced by endothelial and glial cells in retinas with ischemic angiogenesis [32]. MCP-1 recruits monocytes, memory T cells, and dendritic cells to sites of tissue injury and infection [33], [34], and its upregulation may stimulate the infiltration of inflammatory cells into eyes with vitreoretinal disorders.

IL-6, IL-8 and MCP-1 have been independently reported to be regulated by nuclear factor-kappa B (NF-kB) [32], [35]-[39]. NF-kB is found in almost all cell types and is involved in cellular response to stimuli such as stress, cytokines, free radicals, ultraviolet irradiation, and bacterial or viral antigens, in addition to its central role in immune response [40]. On the other hand, VEGF is upregulated by hypoxia through hypoxia-inducible factor 1 alpha (HIF-1α) [41], which is another transcriptional factor that regulates genes which response to hypoxia [42]. As proposed in our study, VEGF may act in an independent pathway to promote the pathogenesis of all the analyzed vitreoretinal diseases, although additional studies are required to completely solve this mechanism. The difference in activation level of a transcription factor may determine the severity of ischemic, angiogenic, and inflammatory changes in ocular milieu.

Banerjee et al. [18] have reported that IL-6, IL-8 and MCP-1 were detected in vitreous fluids from patients with PDR, PVR, idiopathic choroidal neovascular membrane, chronic uveitis, and lens-induced uveitis (LIU). Furthermore, they reported that a LIU patient who went through a complicated phacoemulsification cataract surgery, had the most active disease with higher concentrations of IL-6 and IL-8 than in chronic uveitis or PDR. Combined with our results, the data indicate that the major three factors (IL-6, IL-8 and MCP-1) are critical in multiple vitreoretinal disorders including uveitis. In their report, however, except of LIU, the number of samples in each individual disease were less than ten, which significantly limited the performance of comprehensive analysis between several soluble factors.

Because DR (DME and PDR) and RVO (BRVO and CRVO) are characterized with ischemic retinal angiogenesis, it is reasonable that VEGF participate in their pathogenesis. Interestingly, the patients with PDR/CRVO showed increase of VEGF, but not DME/BRVO which had a significant increase of IL-6, IL-8, and MCP-1 (Figure 1). Nevertheless we do not suggest that VEGF has no contribution in DME/BRVO since the local levels of VEGF in the ocular tissues other than the vitreous may still be elevated. In fact, there are many reports about VEGF as a main exacerbating factor in DME [43], [44].

Another important point concerning DR/RVO, is that VEGF significantly correlated with the major three factors in PDR but not in CRVO. The reason for this may be an insufficient reliability of the correlation analysis due to the small number of samples from CRVO patients. However, in contrast to PDR, all p-values (VEGF/IL-6, VEGF/IL-8, VEGF/MCP-1) in CRVO were more than 0.5 which indicates no correlation (Figure 3 lower three lines, 4B). The lack of correlation may be a result of extremely high concentrations of VEGF in 3 out of 13 patients (more than 1×103 pg/ml) with no consistent high concentrations of the other three factors (IL-6, IL-8, and MCP-1). VEGF is produced from various types of retinal cells including retinal pigment epithelial cells, pericytes, endothelial cells, Muller cells, and astrocytes [45], [46]. Both ishemia and inflammation can initiate VEGF production.VEGF production can be induced by other factors and at the same time initiate a cascade of other factors. Our hypothesis is that IL-6, IL-8, and MCP-1 in the vitreous cavity promote vascular permeability that causes DME. Retinal ischemia leads to an excessive production of VEGF that in turn causes further progression of DME to PDR. On the other hand, a substantial amount of VEGF can be initially produced by the sudden profound retinal ischemia, which in turn induces the major three factors afterward (Figure 6).

Although RRD causes severe visual impairment, it does not induce inner retinal ischemia, which makes RRD a distinct disease from the other examined vitreoretinal diseases. It should be noted that the major three factors were increased unrelated with VEGF in RRD patients (Figure 1). Up to this point there have been reports on significantly high levels of MCP-1 in the vitreous of PVR (a major complications of RD surgery) patients compared to samples from patients with a macular hole or idiopathic premacular fibrosis [14], [47]–[49]. Nakazawa et al. [50] demonstrated that MCP-1 plays a critical role in mediating RD-induced photoreceptor apoptosis. Chong et al. [51] showed that IL-6 is a photoreceptor neuroprotectant in experimental model of RD. It is possible that factors induced by retinal detachment may have an additional function other than inflammation. Additional studies will be required to elucidate this point.

In conclusion, multiplex bead analysis enables a comprehensive analysis of several soluble factors in samples from patients with vitreoretinal disorders, using a small volume of vitreous fluid. The three factors: IL-6, IL-8, and MCP-1 were found to be commonly upregulated and contribute to the formation of various vitreoretinal diseases. VEGF may serve as an additional exacerbating factor in the progression of PDR, and an independent exacerbating factor in CRVO. Moreover, IL-6 and MCP-1 were prominently significant factors in the pathogenesis of RRD patients. Developing a Luminex base technique for the identification of immune mediator profiles in the vitreous opens up new possibilities of characterizing vitreoretinal diseases and designing therapies based on these unique correlations.

## Materials and Methods

### Study Population

Consecutive 339 patients underwent a pars plana vitrectomy (PPV) at Kyushu University Medical Center (Fukuoka, Japan). Only patients recruited from September 2005 to February 2007 were enrolled in this prospective study. Patients profile is shown in Table 1. DME was defined as DR with swelling of the retina caused by leaky vessels which can be detected by either ophthalmoscope/optical coherence tomography (OCT). PDR was defined as DR with obvious neovasculization with or without proliferative tissue. Macular edema complicated by BRVO or CRVO was considered as the decisive indication for PPV. As a control, we selected total 81 patients with either MH or ERM that were free of major pathogenic intraocular changes.

### Vitreous Fluid and Serum Preparation

Under either general or topical anesthesia, undiluted vitreous fluid (200–900 µl) was first collected by 3-port pars plana vitrectomy using a 20- or 23-gauge vitreous cutter with a 5 mL-syringe, followed by irrigation from infusion port. Samples were immediately placed in sterile 1.5 ml polypropylene tubes on ice and stored at −70°C until used. Samples with obvious bleeding were excluded.

Serum samples were also obtained from patients. The samples were aliquoted and stored at −70°C until use. However, not all of them were stored in a well condition. We thus need to reduce the numbers (control: n = 83→53, PDR: n = 147→66, CRVO: n = 13→8), but we could measure the serum concentration of IL-6 and VEGF by standard ELISA technique. For comparison between serum and vitreous, vitreous IL-6/VEGF concentrations of corresponding individuals were extracted from previous measurement (shown in Figure 1), and then reorganized as Figure 5 and Table 5.

The research followed the tenets of the Declaration of Helsinki and the internal Ethics Committees of Kyushu University which approved all the protocols. Written informed consent was obtained from all enrolled patients.

### Protein Analysis and Antibodies

To minimize interfering of fibers and gels in the samples, vitreous samples were diluted 1∶10 in PBS. The concentration of cytokines, chemokines, and growth factors in vitreous specimens were measured using a microbead-based ELISA system [17]. Briefly, in this technique, microbeads with defined spectral properties are conjugated to protein-specific antibodies and added along with samples (samples include protein standards in a known concentration, control samples, and test samples) into wells of a filter-bottom microplate. This mixure is incubaed for 2 hrs to allow antibody and protein binding. After washing the beads, protein-specific biotinylated detector antibodies are added and incubated with the beads for 1 hr. Then after removal of excess biotinylated antibodies, streptavidin conjugated to a fluorescent protein: R-Phycoerythrin (Streptavidin-RPE), is added and incubated for 30 min. After washing of unbound Streptavidin-RPE, the beads are analyzed with the Luminex® 100 (Luminex, Austin, TX, USA). By monitoring the spectral properties of the beads and the amount of associated R-Phycoerythrin (RPE) fluorescence, the concentration of one or more proteins can be determined. The following antibodies were used: Human Cytokine Ten-Plex Antibody Bead Kit, Cat. No. LHC0001, Human IL-17 Antibody Bead Kit, Cat. No. LHC0171, Human Chemokine Five-Plex Antibody Bead Kit, Cat. No. LHC0005, Human Growth Factor Four-Plex Antibody Bead Kit, Cat. No. LHC0004; BioSource International, Camarillo USA). Serum samples were diluted 1∶5 in PBS, then IL-6 and VEGF concentrations were measured by ELISA development kits (Human IL-6 DuoSet and Human VEGF Duoset, R&D systems, Minneapolis, MN) according to the manufacturer's directions.

### Statistical Analysis

All analyses were performed with GraphPad Prism 4.0c (GraphPad Software Inc., San Diego, CA). A non-parametric Mann-Whitney U-test and Kruskal-Wallis test for non-normal distribution were used to analyze immune mediators and patient age variance, respectively. Correlation studies were performed by Spearman's non-parametric test. P-values less than 0.05 were considered as significantly different.
